# On Suicide

**Published:** 1858-07-01

**Authors:** 


### Art. II.—ON SUICIDE.

If it were possible to be satisfied with the popular notion of suicide—"that self-murder is sometimes committed without any sign of mental disease, and sometimes with symptoms indicating such malady"—there would not be any necessity to investigate the subject. But we contend that the occasional verdicts of coroners' juries furnish the best proof that there still exists much more ignorance in the mind of the community, on the *mors voluntaria,* than, for the sake of humanity, ought to prevail.

Cicero, looking with a philosophic eye on the subject, seems to have regarded the fact, that no one could be sane who was guilty of self-murder.* A careful examination of his ideas will clearly show that he had formed a sound opinion as to the improbability of suicides being in a normal condition of mind; for he truly says, "The first thing we should understand is, that every animal loves itself, which, although it be a proposition not to be called into doubt," &c. "How," he continues, "can it ever be understood or imagined for a moment that there can be any animal that hates itself? for contraries would thereby arise, when that desire of the soul arises advisedly to take something to itself which is hurtful to itself; when it does so for its own sake, *it must both hate and love itself at the same time, which is impossible.*" And yet such a contradictory view of the subject must be obvious, when the different verdicts of coroners' juries are recorded. By their decision, we must understand that there exist cases of self-destruction in which the perpetrators have, with a species of *prepense* against themselves, without any sufficient real or imaginary cause, committed suicide, and for this reason they record the verdict of self-murder (*felo-de-se*); and these supposed deliberately guilty personages were, in consequence, buried in unconsecrated ground, or without any burial service. On the other hand, when there has been unmistakeable evidence of mental aberration, and some presumptive evidence that there existed actual disease of the brain, then there is pronounced a verdict of "temporary insanity."

We shall, however, submit evidence to prove that in all suicidal

* "Primum est intelligamus omne animal seipsum diligere . . . . . quî potest intelligi, aut cogitari esse aliquod animal, quod se oderit? . . . . . necesse est quidem, si quis sibi ipsi inimicus est, eum quæ bona sunt mala putare, bona contra quæ mala: et quæ appetenda fugere; et quæ fugienda appetere . . . . sed alii dolore moventur, alii cupiditate, iracundia etiam multi efferuntur: et cum in mala scientes ruunt, tamen Se optimè Sibi consulere arbitrantur."—Cicero *de Finibus Boni et Mali,* lib. v. par. 17, p. 147, c., &c.; Edition de la Reviere, 1606, small folio.

cases there is an abnormal condition of the mental faculties, and that when there is not any manifestation of actual disease, there may be certain indications of functional derangement of the brain, or great visceral disturbance, producing sympathetic disturbance of the intellectual and moral attributes. Suicide can never be committed when the mind is perfectly healthy, as in such condition the strong instinctive love of life tends to render men careful of injuring themselves by the infliction of pain; and, therefore, when the attempt is made to destroy life itself, by whatever methods it may be effected, this can never take place in those who are actually sane. There is not anything startling or novel in this proposition; and we shall endeavour to confirm the soundness of it, by showing that the predisposing causes of suicide are similar to those which induce the various forms of insanity—namely, ungovernable passions, as disappointed ambition, excessive pride, the love of fame, and the irritation experienced when it is not realized; from sudden reverses of fortune; from grief, or anxiety, or strong apprehension of danger; from fear, despair, and melancholy; from unmerited disdain or contempt; and from jealousy, envy, and hatred. It may also be induced by imperfect or confused religious perceptions. If these and other exalted conditions of the mental faculties are manifestations of their abnormal states in acknowledged forms of insanity, and if they also are proved to co-exist in suicidal cases, how can we contend that, in the first instance, the disturbed mental functions are indicative of disease; and, in the second, that they sometimes originate in mere waywardness, without any abnormal state of mind? The very fact that self-destruction is diametrically opposed to a strong instinctive law of our nature—the law of self-preservation—warrants the inference that there are not any suicidal cases which occur when there exists perfect sanity. To comprehend the phenomena, we must test them by the admitted laws of physiology and by the accurate inductions of psychology, even should we seem to run counter to popular prejudices, but which, on the contrary, will prevent much of the confusion which still exists on the subject.

Every one who had observed and thought, would admit the proposed means of testing the data as being the most suitable to render the investigation of practical importance; for instance, in cases of sudden suicide there is evidence of some disturbing influence, which had more or less affected the bodily or mental health, whilst in others, which had been long premeditated, these conditions have been still more obvious. It is therefore essential for accuracy that we trace the various morbific agencies, physical or moral, which have induced some change in the mental constitution. This must be done by a rigid inquiry into the personal history of each self-murderer; and by these means we may learn to distinguish the difference of a series of remote causes from those which had seemingly been the immediate and exciting cause for the commission of the act.* We therefore think that, for the purpose of classification, the views of M. Esquirol are worthy of consideration. He regards the two forms of suicide as *acute* and involuntary, or as *chronic* and *prepense*. This view distinguishes between those cases of suicide which have been long meditated on, and those which have been prompted by some sudden impulse. In the first kind, the act may be induced from some strong passion either indulged or suppressed, which had insidiously affected the organization of the brain; or the desire may be long fostered, from some undefined impression that the death of the party will be of some advantage to the family, or to their faith, and so forth; whilst the latter may act from a temporary depression, either from some real or imaginary suffering, and decide suddenly and promptly to destroy himself, as a lesser evil than the one he seems to experience. It is, therefore, of some importance to endeavour to understand how, under any circumstance, a man can so "*hate*" himself, and pervert a *strong* law of his organism, unless that the change of his nature, which had so perverted his judgment, is admitted to be the result of some morbific process. Had he been in a perfectly healthy state of mind, he would have had clearer perceptions of his duty, and better judgment under any especial exigency, instead of the distorted consciousness which made him assume a right to take away his own life.

In suicides, therefore, we may remark, that it is with them as it is with those who are pronounced insane,—that their opinions would deserve attention, if their premises were realities. But as much of what they state is merely assumed, or, if based on distorted notions of things, is highly exaggerated, so their inferences are as a consequence illogical, rendering it manifest that there exists some functional disturbance of the feelings or moral sentiments; and thus the suicide, like the insane, may also have some organic lesion of the brain, which tends to induce in both cases some form of mental derangement.

Sometimes the two conditions co-exist in one and the same individual, when, for instance, after suffering for years from "a mind diseased," the patient will suddenly destroy himself, from his painful monotonous existence; or when labouring under

* We knew a gentleman fond of horse-racing, who stimulated to great excess without appearing to suffer much permanent consequences, and, as he was a prosperous man, he seemed to be an exception; yet on one occasion, having lost many thousands at a St. Leger, at Doncaster, he left afterwards, and that same night destroyed himself. The long course of inebriation had affected the brain, and under the first strong shock the balance of power was lost, and he acted as a madman!

religious mania, haunted by the notion that he has a call from this world by the agency of some supernatural influence.

Other instances of the proximity, if not identity, of suicide and insanity might be given, when life is voluntarily sacrificed from a conviction that the mind is in an incurable state of disease; and in those cases, when the lunatic has been cured, and is haunted by the dread of a probable relapse, and under this painful impression puts an end to his existence, and also in long-standing cases of melancholy, there is often manifested a strong determination to destroy themselves.

Whatever may interfere with the actual healthy condition of the mental faculties, whether the cause is idiopathic or sympathetic, may induce either insanity or suicide. Under such conditions of mind, the mental vision becomes indistinct and gloomy, and, like children in the dark, they conjure up frightful images, and are startled at their own creations, when sometimes the shock to the nervous system may end in insanity; or, to get rid of such morbid impressions, they commit self-murder, their mode of effecting this act of desperation being influenced by some means which, in ordinary parlance, are accidentally presented to them when they have decided on the act!

We will cite a few instances in illustration of the previous statement.

A. B., a Wesleyan, had been in a state of mental depression for some time, arising, it was said, from some religious misgiving as to his spiritual state; but still he attended to his business. On the morning on which he destroyed himself, he had been to chapel, when something in the sermon he applied to himself, and was more than usually taciturn as he returned home with his wife and family, but still not sufficiently so to excite either alarm or suspicion. He said that he wanted to go into the shop to get a tract he had left there, when it is supposed that, seeing a large knife which he had used in his business, this suggested his determination. He went up into his bed-room, carrying the fatal weapon with him. There he carefully placed his Sunday coat and waistcoat on a chair, and then nearly severed his head from his body.

A parish clerk of C——, who had in a comparatively short time lost his wife and family from typhus fever. He became melancholy from this sad visitation, and attributed it to his own sinful habits of inebriation. But instead of becoming a more sober man, he continued to drink to excess; and although his sister (who acted as his housekeeper) was warned not to leave any weapon in his way, and which she prudently attended to by taking away his razors, and every article of dress which might have been used for strangulation, yet one morning, as he had not made his appearance at the usual hour, his bed-room was entered, and he was found drowned, having suffocated himself in a chamber utensil.

A pensioner at B—— became melancholy from the presumed incontinence of his wife. One day, after he had been cleaning a horse-pistol, he loaded it. His wife came into the room soon afterwards, when he charged her with his morbid suspicion. She upbraided him, and told him if he were not drunk he would not have dared to say anything so insulting to her; and she said, on leaving him, that he was a most contemptible coward. Whether there was a feeling of remorse for making such a charge—of which he had no proof—or the word *coward* grated on his ear, or whether he regarded it as a taunt for bearing his supposed infamy so tamely, it is impossible to say; but the pistol, already primed, was near him. This he seized on, placed it in his mouth, and fired, shattering his skull, and dashing his brains up to the very ceiling!

There are many other conditions of mind, besides those already noticed, which induce self-destruction; and none more fatal than that restless state designated "*tedium vitæ*," when the mind seems incapable of being healthily occupied. There is then experienced a desire to leap the gulph which separates the living from the dead. When such a condition of wasted energies exist, the irritable and strong nervous irritation must be regarded as a diseased condition of the mental faculties; for, as it is an imperative law to preserve the normal functions of the bodily organs, so that they should be exercised, it is equally so in reference to the mind—lassitude and diseases are superinduced when these sanatory conditions are neglected.

We have many well-marked instances of persons with a plethora of wealth, who had been pampered from their infancy, and allowed to take their own course as to their mental culture; when soon the injudiciousness of this treatment has been obvious, in the waywardness and fickleness of the individuals, and from a feverish desire for change, which have brought neither pleasure nor satisfaction; and having had neither object nor purpose in their existence, they have, under a temporary loathing, destroyed themselves, in the same wanton and purposeless manner that a spoiled child will suddenly break a toy he has been importunate to possess.

Persons of this class, which belong to the acute or involuntary form of suicide, according to M. Esquirol, present many phases. There may also be included in this division those who, under sudden remorse, often, when in a maudlin state of inebriety, commit self-murder; and those who, under a sickly condition of the moral sense, after suffering from a debauched career, have recourse to the same quietus. Others will destroy themselves when offended by some insulting phrase by a superior, or when treated with contumely by an equal. Yet this very sensitiveness is the sign of positive functional disease. Women who have been seduced, and afterwards treated with scorn and insult by the heartless wretches who have accomplished their ruin, will, under the twofold effect of pained affection and remorse, destroy themselves. So also will timid and excellent men who dread any threat of criminal proceedings, even when they are guiltless. All these different victims of a state of mind which resembles, if it is not actual insanity, may suddenly, in a moment of terror, remorse, or any other outraged feeling, precipitate themselves into a river, or cut their throats, quaff poison, hang themselves, and so forth, merely to rid themselves of the irksomeness and irritation under which they suffer. As a general rule, their intellectual faculties possess neither power nor intensity; but there are, on the other hand, too many exceptions of philanthropists and able jurisconsults who have put an end to their existence, although, if such men had had healthy minds, they would have endured their allotted trials.

There is another phase in the acute form worthy of a passing notice. We allude to those who have exercised their minds with great activity for many years, and when they have obtained the *otium*, will seek for the *dignitate* in some arcadia, where, amidst plenty, they expect to realize their long-wished-for rural happiness, and a calm and quiet existence—this state had formed the staple thought of their waking dreams, whenever there was a lull in their previous laborious occupations. But, alas! torpor is the symbol of death, as activity is that of life; and so they find their long-cherished hopes a mere phantom, an "*ignis fatuus*," and they soon tire of their meretricious splendour. The very quietude renders their listless lives so much more monotonous, until it becomes too irksome for endurance, and then, under a sudden impulse, they either destroy themselves, or become melancholy and hypochondriacal. Numbers, therefore, have finished a life of constant industry in the asylum, or by their own hands. And by their death they have bequeathed an admonitory lesson to avoid either excessive mental labour, or so little that inanition results, and destroys, in either case, the harmony of the mind.

In all *chronic* cases of suicide there is evidence of a gradual perversion of judgment, and with so much disturbance of some of the feelings or sentiments as to render it a matter of legal induction, that if not insanity, it so resembles it that it must be regarded as a phase of mental disease. In many instances it may be difficult to trace the actual predisposing cause, when the fatal act has occurred; yet, if the manners and conversation of such persons were carefully noted, they would have manifested some change from their ordinary mode of acting, or some perverted perceptions indicative of moral inconsistency. If the friends, under such conditions, had consulted a physician, he would have detected some defectiveness of their consciousness, and have suggested precautionary measures. But in the social circle such changes of temper or disposition are considered mere wantonness or caprice,—instead of assuming the actual truth, that such discrepant manifestations from the ordinary habits of thinking and acting were, in point of fact, premonitory warnings of some diseased condition of brain; just as coughing is indicative of some incipient disease of the lungs, or the mucous surfaces, or the air-passages. Remedial means would relieve both by timely application, when the symptoms may have resulted from functional disturbance; but if allowed to proceed until some lesion occurred, and the organic condition of the brain became implicated, the mad-house or self-murder must be the inevitable consequence.

We will now allude to those inveterate cases of suicide which assume the *chronic* form, when all the precautions to prevent the act may be abortive, and the long-premeditated end is persisted in until it is consummated. Often when saved after a first or second attempt, the infatuated being will persist until the act is finally accomplished. As an instance which made a deep impression on us, we relate the following:—

Mrs. B—— was the widow of an officer, who had married her for her beautiful person. She had been a servant, living with a family whom Captain B—— visited; and though there was a great difference in their respective ages, and in their education, he proposed to marry her, and was accepted. But in order to fit her for the rank to which he had removed her, he had her taught things of utility, as well as some accomplishments, and they seemed to live very happily. After a time it was currently reported that she had formed an illicit amour with the man-servant, and that, in consequence, her husband had manifested a strong dislike to her (one of the results of intense jealousy or of violated confidence); but whether the scandal in either case had any foundation, it is impossible to decide. Nevertheless, one part of the report seemed correct,—they did not live so harmoniously as they had previously done. The captain had a short and sudden illness, and was gathered to his fathers, attended with military honours. His widow covered herself, not with sackcloth and ashes, but deep crape, and seemed greatly affected at the loss of such a good and true man. He left her independent, but not rich, and she continued to live in a respectable position. Soon after this event the man-servant married, and Mrs. B—— began to droop, and became melancholy; and the gossips affirmed that she was piqued that her paramour had forsaken her, and that her mental disease arose from the chagrin of offended vanity, and some remorse that she had sinned against the man who had treated her so generously, and with so much consideration. Instead of rallying, her mental sickness assumed a more chronic form ; and one day our quiet and respectable neighbourhood was startled by the report that "Mrs. B—— had cut her own throat!" Knowing her medical man, some of our family inquired as to the correctness of this statement, and he confirmed its literal truth. The wound was not mortal, but she was weak from loss of blood, yet it was thought she would recover; but a nurse was placed to watch, lest she should attempt to tear open the wound. After a few weeks we saw her, pale yet beautiful, and with such a melancholy expression as would have rendered her a charming model for a Magdalen. Months passed away, and she seemed, if not happy, yet reconciled to life. She renewed her pastimes, and occasionally saw company. Yet when it was supposed that she had recovered her mental health, we heard that she had hung herself! The servant, who found her suspended, cut her down and sent for a medical man. She was again restored to life, when she expressed her deep regret that she had been guilty of such an act.

Her remorse must have been the result of some real or imaginary cause ; for about six weeks after the latter circumstance, she went out one morning very early, dressed in a riding-habit, but not returning to breakfast, a hue-and-cry was raised, and after a search of some hours, she was discovered drowned in a mill-pond. Though dead, she looked beautiful. Her long brown eyelashes, though closed, and her rich profusion of the same coloured hair, contrasted well with her dark-green habit, and gave her the appearance of one in a calm sleep ; but in her hands there was grass, which she must have seized from the bank, as if there had been some sudden return of healthy consciousness, and a desire to prevent the death she had so perseveringly sought. The latter may have resulted from the sudden plunge into the cold water, and the tonic effect it temporarily produced on her brain ; but she was too much entangled by her dress to save herself, and the current was so strong that she had drifted a long way from the place where her hat lay. The verdict of the jury was "Temporary insanity."

In this case, however imperfectly sketched, there is sufficient data to trace the predisposing causes of her morbid condition. Any woman who had yielded to her prepossession in favour of one in an inferior grade, would have both her affection and vanity pained if he proved worthless and untrustworthy. In Mrs. B——'s case, she had been neglected by the very man to whom she had sacrificed honour and conjugal duties; and there was, besides, "the still small voice" that taunted that she had sinned too deeply even for repentance, as she could not now compensate for the injury she had done to her excellent husband; and young and beautiful though she continued, her remorse was so great that she loathed her existence! Yet no one would affirm, if this history of the causes which induced her mental condition is correct, that she was sane when she persisted in the fixed idea of destroying herself. The evidence submitted is important, as showing how the balance may be disturbed in a sensitive mind, when the moral sense becomes morbidly affected; and we have some clue to explain the whole change in her mental constitution, and the derangement which subsequently supervened—a derangement which tended to subvert the instinctive love of life, and to induce a constant desire for its annihilation. We might say, that even when a verdict of "Not proven" is substituted, so far as the *actual* guilt of such a being,—yet the whole history of the case, and the catastrophe, renders it a matter of presumptive proof that there was some painful state of the moral sentiments, which had quite metamorphosed one fitted for healthy enjoyment to seek for death in the midst of more means of happiness than she could ever have anticipated.

We will now give a case, when a similar result ensued, not from any moral delinquency, but from a threat to prefer a charge which would fix a stigma on one whose character was unblemished. We quote the facts from the narrative portion of a lecture delivered by E. P. Hurlbut, Esq., before the Mechanics' Institution of New York, "On the Legal Protection of the Sentiments and Feelings." After many apposite and sound views having reference to his especial subject, the lecturer said:—

"Many of you, doubtless, remember a case of suicide by a young gentleman in this city, some two years ago, who rushed to the top of his house, which was three stories high, and precipitated himself thence upon the pavement below, thus occasioning his awful and instantaneous death. A few weeks before this melancholy event he was in perfect health, mingling with fellow-citizens, having their highest respect, and the attachment of many warm and devoted friends. His domestic character was a model of the most affectionate kindness and perfect devotion to the happiness of his mother (his only surviving parent) and his brothers and sisters. His charities were liberal; no worthy applicant for aid went away empty from his door. He was generous to a fault. His integrity was of the highest order, and he preserved the most unsullied honour; it was his soul—his life. In fine, he was one of the noblest young men I had ever known, and one whose memory I shall always cherish to the latest hour of my life. I would it were divested of the story of his unhappy fate!

"A few days before his melancholy death he called upon me, under great excitement of mind, and stated to me, more as a friend than as his professional adviser, the details of a conspiracy formed by several abandoned people in this city to extort money from him, one of whom had sought his acquaintance to ask charity, and who had received pecuniary relief at his hands. The conspirators had a scurrilous paper in their interest, and a threat of a libellous publication had been made in its columns. This was his concern at the time of his visit to me. I inquired into the whole matter with great interest and anxiety. I know the truth of his case, and I know to a moral certainty that there was not a shadow of a just foundation for the least censure on his fair fame. I advised his treating the conspirators with utter contempt, and to pay them not the least attention. He soon after received from some lawyer, who read the laws but to violate their spirit, and whose moral nature was attuned to the work of mischief, further intimation that the conspiracy was to be further consummated by a suit at law. He brooded over the matter till sleep and rest forsook him. The scurrilous print came out with its brutal libel, and its victim fell beneath the stroke.

"When he next called upon me, which was the day after publication, *I think his whole appearance was that of a maniac; and his wild exclamations, his intense mental suffering, amounting to the most dreadful agony, baffled description.* Alas! I could not soothe his wounded spirit; he was taken to his home, and when inquired after the next opportunity, I learned his death. This man was murdered, and the murderers live unmolested by the law."

The lines marked in *italics* are ours; and we have not any hesitation to say, that had this excellent young man, whose moral susceptibilities were so fearfully deranged by a foul libel, consulted a physician instead of his legal adviser (though an estimable and intelligent man), some means would have been suggested, and the horrid catastrophe might have been prevented.

Cases of the acute form of suicide, as M. Esquirol would have designated the one given by Mr. Hurlbut, are rare in comparison with those which assume a more chronic form; and yet such acute and sudden development of the suicidal tendency furnishes the clearest psychological evidence of functional disturbance of the mental constitution, indicated by defective ratiocination' and a derangement of all the moral perceptions. Like in monomaniacs, they have the one idea which haunts their imagination, and death to them is the only means of exorcising it. In our experience we have remarked that such persons are of a highly sensitive organization, the intensity of which is greatly increased by their nervous or bilious temperament. Examples have been already given that the predisposing causes may be sudden pecuniary embarrassment, real or fancied jealousy, or the dread of infamy, and so forth, and which, by arousing into fearful activity some of the affective faculties, destroy the mental harmony, and then in a fit of desperation life is sacrificed. In Mr. Hurlbut's case, a man of pure mind and life was so disturbed by threats which he supposed would, though innocent, still stamp him with ignominy, that his existence became to him intolerable. Even in such acute forms, when the suicidal act is perpetrated, there is unmistakable evidence of positive insanity. Such disturbing influences might occur to individuals of merely a lymphatic temperament, although the process would be more tardy, but ultimately similar consequences might result. An individual of this kind would brood over the source of his irritation, and gradually affect the healthy action of the brain, and then his life's drama might suddenly terminate in an act of apparent deliberate self-destruction.

In both the acute and chronic forms of suicide, the mind is clearly in an unsound state—an opinion which has already been advocated in this and other journals. The cases of suicide which have been regarded as exceptional, are those wherein the act has been committed with apparent deliberation, and with a coolness worthy of right and praiseworthy objects: such as when life has been voluntarily sacrificed under the impression of an incurable disease, and a deliberate determination to avoid a continuation of the suffering experienced. But persons who have committed suicide under such circumstances, have reasoned contrary to all sound data, and in violation of the strong instinctive feeling of self-preservation. It is true that they may have had their perceptions sympathetically perverted by the fixed sensation of fear with which they are impressed, and would not be regarded as actually sane by the competent physician who had studied the effects of suffering on the vital organs, and the disturbing influence of continued pain on the organs of thought and feeling. So, also, although there are cases on record when persons under pecuniary difficulties have destroyed themselves, apparently with great coolness and deliberation, yet this should not be regarded as any proof of their sanity, inasmuch as all who are in embarrassed circumstances do not destroy themselves. Hence the inference must be, that when they do so they act insanely, being predisposed, from some defect of their organization or education, to distinguish what are the legitimate acts of a man's volition, and what are interdicted in the very constitution of the mind in its relation to the Divine Author.

The following case, which induced a jury to pronounce a verdict of *felo-de-se*—as, in their judgment, the voluntary death was the act of a sane man—is cited to show that even the evidence on which that opinion was founded, furnished indubitable testimony to the contrary. We are forced to quote from memory, having mislaid the newspaper (published at Chelmsford) containing the report:—

"Some years since, a gentleman of the name of D— arrived at Saffron

Waldon, in Essex, in a post-chaise, from Dunmow, and drove up to the principal inn. He was by profession a surgeon-dentist.

"The morning after his arrival he called on some of the medical men, who, finding him a man of cultivated mind, and with a respectable knowledge of his pursuit, promised him their support. He intimated his intention to deliver a lecture at the Town-hall, 'On the Natural History of the Teeth.' He therefore issued a circular intimating the evening on which it would be given, and, at the same time, announcing that he might be consulted daily.

"Some of the medical men who attended his lecture were still more satisfied with his qualifications, and pleased with his polite and urbane manners. He is represented as a man with an intelligent expression, dark eyes and hair, a blond colour, with an aquiline nose and good mouth, and that he made a favourable impression on his auditory.

"He gave little trouble at the inn, and was rather abstemious in his habits. On the Saturday, just a week after his arrival, he was observed to be very moody; but still this did not produce any unfavourable impression, much less any suspicion as to the actual condition of his mind. On the contrary, it was attributed to his ill-success, as he had had but one professional visit.

"The next morning, as he did not make his appearance for his breakfast, some misgiving was felt, and the waiter went to see if he were indisposed. As he received no answer when he knocked, he tried the door, and found it locked. A forcible entrance was made, and the poor fellow was found dead on the floor, lying in a pool of blood, as he had effectually severed the carotids with a razor!

"It was then remembered that he had been heard pacing about his room for some time after he retired, during which he had taken the precaution to destroy every scrap of paper, and to cut out the marks on his linen, as if to prevent all clue to his personal identity. And he had also addressed a letter to the jury, prior to the performance of his last fatal act. This document commenced by stating that he had long battled with misfortune, and his whole life had been a series of impotent struggles; that his ill-success arose from no fault of his own, so that he had no other alternative but death. He then went on to say, that some would deem his act as extreme cowardice; and others, that it was one of daring presumption to his God; that this latter was a mere personal matter between himself and his Maker, and that he believed that the Great Author judged of motives, and that he did not doubt of being pardoned. Then he continued thus: 'This act of mine is clearly an act of self-murder, and the verdict must be *felo-de-se*.' Finally, he spoke of his obligations,—that he had paid up his account to the previous day; and he requested that his instruments might be sold to liquidate the remaining debt, and the surplus to be given to the servants, to whom he had not given any gratuity, &c.

"The jury brought in the verdict he had pronounced on himself. He was buried on the cross-roads near the town, and a stake driven through his body."

In this brief view there is furnished evidence of unsoundness of mind; for with his previous experience of the tardiness of professional application, and with the evidence of his positive integrity, it might have been supposed that he would have avoided the expense of travelling post, and sojourning at an inn; and that if he had been actually sane, that he would have economized his means by taking some reasonable apartments, and thus been enabled to await with some little patience the chance of replenishing his almost exhausted exchequer. So much for the facts which ultimately induced him to commit suicide. But there are other moral aspects which may be worth noting. He seemed to lack moral courage, or any firm reliance on Providence, otherwise he would have struggled on even had his prospects been still more gloomy.

The jury considered that his letter furnished strong evidence of self-felony, induced by a deliberate criminal intention against his own person; and that his justification, though erroneous, was a proof of his sanity. For our part, we should decide the contrary by the presumptuous and contumacious manner in which he justified his act. Had he been sane, he could not have spoken with such flippancy of the Majesty of Heaven. It may be said that if this constitutes a proof, then all inveterately sceptical persons should be regarded as lunatics. If they are not so in the eye of the law, they certainly indicate proofs of a want of harmony of the mental faculties; and we must decide in the case of D——, that he indicated some defective conditions of the religious perceptions, either from some defectiveness in his mental constitution, or by some warping from defective culture. The inference we have drawn as to his actual abnormal state of mind is capable of some further presumptive proof, if we investigate what strong motive might have influenced him in urging a verdict of *felo-de-se*.

We may mention, incidentally, that a gentleman at Saffron Waldon told us "that D—— had a fine Oriental physiognomy, and that he felt assured that he had been born an Israelite." This fact would explain the difficulty, that amidst the distraction and want of confidence resulting from his scepticism on spiritual subjects, there seemed to lurk the prejudice of his people, that if he could not be buried "in the cave of Machpelah," where his ancestors lay, that he would prefer unconsecrated ground rather than lay in the consecrated ground of any other form of religious belief. Now, whether the suicidal act had been the result of long deliberation, or from a sudden impulse, the insanity is obvious from the incoherence of the reason for the act, and that his very conclusions had simply resulted from his incapacity of perceiving the erroneous data of his premises—so that the inferences which had induced the deed itself were absolutely unsound, and positively absurd. If this victim had been actually sane, his crime would be greatly aggravated; for in committing the sin he had seemed reckless of consequences, and had added to his ill-deed the great offence of using insulting language to Him whose law he had determined to violate. We, however, cannot hesitate to decide that his conduct marked one of decidedly unsound mind. This induction is founded on positive data, particularly if we reflect on the predisposing causes of suicide, which are found to be precisely similar to those which induce some of the forms of insanity, so that it would seem in all cases that it is difficult to draw the line of demarcation. In both there is evidence of some disease of the mental faculties.

If we were to indicate any particular form of the affection by which to specify the perfect analogy between suicidal cases and insanity, we would instance certain states of the domestic feelings induced by some sudden and strong emotion which tends to make persons so powerfully deranged, that for a time they will "play such fantastic tricks before high heaven" as to give evidence of some morbid aberration of mind, which might be of temporary duration if medical aid had been procured, but which assumes a permanent form of disease if neglected, and then it might end in fits of despair, revenge, murder, or suicide. Nay, there are instances of both crimes being perpetrated at one and the same time.

The following case will illustrate the latter statement:—One night, during our visit at H——, we heard a report that Mr. —— had murdered his wife, set fire to his house, and then killed himself. We went with some gentlemen to visit the tragic scene. The premises had been fired at different places, in an apparently deliberate manner. In the bed-room, in which lay the murderer and suicide, we found the bed-curtains burnt to tinder, presenting a crape-like mourning where the innocent victim lay, through whose head a pistol-ball had penetrated the brain. She was lying in a pool of blood, whilst the corpse of her husband was stretched on the carpet, cold and stiff, with a pistol close to his temple!

The circumstance itself excited great commiseration for the murdered lady, as her death seemed to have been the result of previous deliberation. This was confirmed by the evidence at the coroner's inquest. It was proved that on the previous Saturday the suicide had sent two poisoned loaves to his sons, who were at a boarding-school; but, in consequence of the tragedy taking place so soon, information was forwarded to prevent their being eaten. It was also stated by one of the servants, that, on hearing the pistol fired in her mistress's bed-room, she took a light to ascertain what was the matter; that she spoke to her mistress, but did not receive any answer; and that then she beheld the curtains in a blaze. In the midst of her terror, and at the hazard of burning herself, she took means to ascertain if her mistress was there; and just as she obtained proof of the fact, and began to make an alarm, her master rushed in like a fury. She fled, and he fired at her, but missed; and she had scarcely regained her own room, when she heard another report, and a heavy fall. All the servants screamed, and rushed to the street; their screams, and the appearance of fire, brought immediate assistance, when the latter was put out, and the horrid affair was then revealed. The whole town was in a state of excitement, and every one marvelled what could have been the cause for such a series of unnatural and savage acts.

Mr. —— was reputed to be a rich merchant; was a professor of religion; and lived, to all appearance, on terms of affection with his wife and family—so much so, that it was observed that, when he went for a morning walk, or to church, some of his children usually accompanied him. Besides, he was a man of gentlemanly bearing, seemingly kind in his manners, and generally punctual in his engagements. But, on investigation, it was discovered that his affairs were embarrassed; he had two establishments to keep, and two families to clothe and feed; for it appeared he had had a paramour with whom he cohabited, either before or soon after his marriage. To understand, therefore, the probable condition of his mind prior to the final catastrophe, we must reflect that he was not like a common, vulgar, uneducated man; he was well versed in his relative duties to his family, to society, and to his God; and he could not blink the sinfulness of his career, and the despicable acts he had to commit in order to conceal his immoral conduct;—then, again, his affairs had become so involved, that he could not continue to ward off the infamy of his conduct. These things, conjointly, must have fretted him greatly, and affected his moral sense, whose "still small voice" must have continually tortured him, and denounced his follies and his crimes,—and thus he was ultimately driven mad. For one of the strongest collateral evidences of his insanity is manifested by the fact that, whilst he endeavoured to destroy his legitimate children and his wife (a most charming woman), he did not try to injure those who, in the eye of the law and in the judgment of society, had not similar claims on him.

Who could pronounce Mr. —— to be sane? He had placed tallow candles in different cupboards, which he set fire to just about the time he had intended to shoot his wife and himself, under the unsound assumption that his whole residence would be burned to the ground, and that then no evidence would exist to implicate him, or to explain the origin of the horrid affair. If his mind had been in a healthy state when he concocted his diabolical scheme, he would have been certain of exposure and infamy; he would have thought, that if the servants smelt fire, or the flames were visible,—and if to these be added the report of pistols, he would have calculated that alarm would be given, and that, as a natural consequence, the double murder would be discovered, and that an exposure would be made of his immoral duplicity, and the desperate condition of his pecuniary affairs.

We have deemed it right to mention these facts, as, by their means, we can trace all the incidents which predisposed the state of his mental faculties, and induced the fearful tragedy we have narrated. His duplicity caused too much strain and tension on the brain, and could only tend to mental derangement. He used to go to the counting-house, and return to dine with his family; but much of the time which should have been spent in business was devoted to his immoral purposes. Can it then be wondered at, that he was in a constant state of irritation? And he must have had an incessant struggle how to rid himself of his moral annoyance, and to relieve himself of his pecuniary liabilities, until at length his own preservation became the all-absorbing subject of his mind. This constant painful excitement made him dwell on the means of avoiding certain infamy; this *one* thought engrossed his whole attention; and it at length manifested so much intensity, that his judgment lost its influence, and his moral sense became so depraved, that it could no longer exert any restraining influence over his conduct. And when the balance of power was thus lost, the selfish idea then suggested no other alternative—infamy or annihilation! In the arrangements he made, he showed an absence of all common sense—a mere muddled state of brain, which defeated his own tortuous policy, and thwarted the intention of the result of his desperate plan. If he had not been a slave or a coward,—made so by his strong desire to prevent the public disgrace that awaited him, should any exposition of his conduct take place,—he would not have attempted to kill his own offspring with poison, murdered his wife, attempted the life of a servant, set fire to his house, and, as a *finale*, destroyed himself.

There is one practical lesson derived from this melancholy and fatal history—namely, that when any of the feelings obtain such despotic influence, there cannot be any sane perception, and self-control is then more or less defective; and then the intensity of the selfish propensities increases inversely, in proportion as the moral sentiments become torpid,—the latter lose all influence over the actions, whilst reason is too feeble to whisper a protest so as to restrain or save the victim of passion, even when the most criminal actions are contemplated. In the case of Mr. ——, there was a feverish condition of mind; he either had not time, or else he could not deliberate on the consequences; and then he was the victim of disease, hurried onwards to the frightful gulf without one thought of the actual danger until he had perpetrated the first deed of violence, and was then driven irresistibly on, and in a reckless and desperate mood consummated the last act of madness.

We think it not improbable that many domestic tragedies are the result of some moral compromise in the first instance, and not always from any strong natural criminal tendency. Whatever, therefore, tends to affect the mind's harmony, is fatal to its health. It matters not what the disturbing influence may be,—if it disturbs the whole thinking process, then it is certain to induce some form of mental alienation. Whatever, then, may become a fixed idea, assumes a palpability, having all the vividness of an actual picture, which so impresses the mental vision, that every other thought is rendered confused and indistinct, until a sensation is experienced that the only way of getting rid of the phantasma is by self-murder. We think, in Mr. ——'s case, the verdict of the jury was "Temporary insanity;" and they were right in saying so, particularly as it was remembered that for some weeks before the sad occurrence there was observed some alteration in his manner. He was more irritable, and less attentive to his toilet; he drank more wine, was more abstracted and taciturn, and betrayed more impatience than was his wont. If these symptoms had been regarded as indicating some abnormal condition of the mental faculties, the miserable result might have been prevented.

In cases where there has not existed any criminal predisposition to account for these forms of mental derangement, yet somewhat similar results may be induced by an over-sensitiveness of the nervous system, often from extreme anxiety arising from great pecuniary difficulties. We could cite some interesting illustrations, where a chronic form of disease has disturbed the mind's sanity. The various agencies may act insidiously, until there is some positive injury to the organization of the brain. Other important vital organs may be implicated, which, reacting on the seat of thought and feeling, aggravate the symptoms in the ratio of their disturbing influence. Dr. Mantell says that—

"During the last twenty-five years many cases of suicide have come under my notice, in which the mental hallucination which led to self-destruction had depended on lesions of the brain, occasioned by slight or neglected injuries of the head, to which neither the patient nor his friends attached any importance. In several instances of self-destruction without any assignable moral cause, and in which no previous signs of fatuity or insanity were manifested, I have found, on a *post-*

*mortem* examination, either circumscribed induration, or softening of the brain, or thickness and adhesions of some portions. The conviction was therefore forced on my mind, that very many of the so-called nervous or hypochondriacal affections, which are generally considered imaginary, and dependent on mental emotions, are ascribable to physical causes, and frequently originate from slight lesions of the brain."

The effect of cold and hunger may induce insanity and suicide, and which fact is vividly described by Baron Larry as occurring on the retreat of the French army after the burning of Moscow. We also know that great misfortunes or deep humiliation to the proud and sensitive when they become the recipients of alms, that this degradation will so affect them, that the whole chylopoietic viscera become disturbed in their functions, and in their turn aggravate the morbid condition of the mental faculties. Thus some painful emotions may so affect the liver, and a state may be induced of morbid depression, and which may for a time exist with symptoms of self-destruction, and yet if cured before the brain is organically affected, the symptoms also cease, and a healthy instinctive self-preservation supersedes the previously painful condition. On the contrary, if the hypochondriac is neglected, under the erroneous impression that the complaint was bad temper, then the mental depression which was in the first instance an effect, becomes a cause, and the suicidal disposition, which had only been a symptom, assumes the more chronic form, and may be persevered in until the act of self-destruction is accomplished.

The latter cases are well distinguished from those sudden manifestations under a temporary irritation of temper, or the lowness of spirits after a debauch, or a quarrel with a lover, and so forth, when often such persons talk of destroying themselves. In the majority of such cases the threat is never seriously entertained. But should these persons, under the impulse of irritation, carry the threat into effect, if the patients are promptly saved, a reaction takes place, and they never repeat the folly or the crime of the attempt; and generally the cure is so effective that not any further endeavour is made to abridge life. These, however, can never be confounded with patients where there exists a strong tendency to self-destruction, and which, from the intensity of the desire, must be regarded as a state of actual disease; such a case as that of Mrs. B——, who hung herself, then cut her throat, and finally drowned herself. For such beings the suggestions of Dr. W. A. F. Brown, "for an asylum for patients recovered after an attempt at suicide," would be highly important, and cures might be made of even such chronic cases, unless there existed extensive lesions either of the brain or its membranes. The following extract from the article is worthy of attention :—

"No one can read the public papers from barren curiosity, or catch the moral characteristics of the time, and shut out a conviction of the frightful increase of suicide meditated and effected." And he regrets "that the frequency of such events, and the publicity given to them, often impart to the suicidal disposition an epidemic or imitative character. We may daily observe it stated in the public papers, that persons who have been prevented from the commission of suicide, are immediately, on their recovery from the effects of the attempt, set at liberty, and allowed to return to their friends and home. This is very questionable humanity. It is, in effect, to deliver unfortunate beings, a prey to their shame, or sorrow, or madness, to the very motives of the act they meditated, and will still meditate, and to these aggravated by exposure and obloquy. Individuals, under such circumstances, cannot be regarded as responsible, or expected to understand so clearly in which they have been, and are, as to resume at once those modes of thinking and feeling on which dependence can be placed, and in which the safety of the miserable beings consists. Assuredly they are neither trustworthy nor rational, and yet it is doubtful whether they can be treated as insane. The law forbids that they should be confined and protected from themselves in an asylum, however appropriate such a retreat may appear for their condition, and however closely connected that condition, when analysed, may be found to be with mental derangement."*

That it should have ever been doubted that there are many symptoms in common between mental derangement and suicide is indeed a matter of surprise. Place all the phenomena of both these forms in juxtaposition, and it is impossible to distinguish the difference of a person with one fixed idea—that of his own self-murder, and the different hallucinations which are actual existences to the mental perceptions of the insane; and that it should be deemed right in the latter cases to protect the individuals from injuring themselves, and to abandon suicides to caprice or accident; circumstances so similar, yet treated so differently, demonstrate the defectiveness of the state of the laws, arising from the fact that the distinctions between insanity and suicide are made rather from the popular views of these different affections, than from the more certain *data* of the physiological and psychological sciences. No one cognisant with medical physics could possibly say that when there exists a fixed determination for self-destruction, that it is not a sign of mental disease, because the very premeditation is opposed to the instinct of the *normal* mind. But we have the testimony of many practical and learned physicians who have given special attention to the subject, and we find that their experience confirms the *a priori* reasoning—that in suicides long meditated there is found, on a post-mortem examination, some organic lesions of the brain, or some altera-

* "Lancet."

tion of its structure; and that when no such signs are indicated, that then the membranes of the brain, or the skull, show some proof of diseased conditions. By the evidence, therefore, of these most qualified witnesses, there is confirmed, what might otherwise appear merely speculative inferences on the predisposing causes of both these kinds of mental affections. When, however, in the positively insane and the determined suicide the brain, &c., does not appear to be implicated, that is, so far as our experience can detect, then in both instances it will be found that some of the organs of vegetative or animal life are in a diseased condition, and by reflex nervous action affect the organ of thought, and induce symptoms of such mental derangement, just as if the mental organs themselves were idiopathically affected.

We have alluded, incidentally, in the extract from Dr. Brown's paper, to the epidemical form of the suicidal malady, but which is a subject in itself so very important, as to demand not a mere passing notice, but an elaborate investigation from more careful statistical tables than now exist. These tables should be collated from well-marked cases of this kind in counties, districts, and countries, in which certain special difference should be noted, not only when they are epidemical, but also when they are endemical.

If it is admitted that there is such intimate connexion between particular conditions of the body and the states of the mental faculties, it is not surprising that, in a low marshy district, for example, where the *malaria* induces low forms of fevers, that in its incipient stage, if one so circumstanced commits suicide, that others suffering from precisely similar depressing sensations, should adopt a similar mode of making their quietus. Having thus alluded to an epidemical fever as liable to induce epidemical suicide, and that this sort of imitation arises from a similar condition of the bodily and mental faculties; we would now also call attention to the fact, that a *special* moral state of a people may exist, which may render them disposed to listen to any suggestion, even as to the best method of how to kill themselves.

About the year 1818 there was published a *brochure* in Paris, recommending ignited charcoal as an easy and agreeable mode of dying. A programme was given of all the details to be attended to, and a lively description of the *modus operandi*, when death was described as advancing in a noiseless way, without either startling the conscience or the consciousness, and that a gentle drowsiness covered the senses, and continued to increase, until the world, and its joys and its sorrows, were oblivious!

The following year, we learn that out of two or three hundred suicides in Paris, one-fifth of these cases had died from ignited charcoal. The imitation in this instance arose from a mental

*idiosyncrasy,* a quasi-philosophical indifference of life, a state often induced by a conception that all the vital phenomena are the result of mere bodily organization, and that mind is merely the result of cerebration, or in other words, a secretion of the cerebrum.

With such opinions men may seem to commit suicide in what, in ordinary parlance, might be called health; but as the condition we have named is in violation of all the teachings of theology, and at variance with the experience of mankind in general; it would seem that the intellectual and moral defect, inferred from the incapacity to appreciate the most ennobling truth, "man's immortality," would be presumptive evidence, if not of positive insanity, according to any acknowledged legal definition, yet of that kind of defectiveness of some of the mental faculties by which scepticism is induced as one of its consequent hallucinations.

We have noticed, in this country, that when some special mode of self-destruction has occurred,—as, for example, taking prussic acid,—that this has become, for a time, the prevailing method of suicide. And lately, we have observed so many cases of throat-cutting, that it seems almost epidemical; and yet there has not, probably, been any acquaintance between any of these infatuated beings. Among the unfortunate, dissolute, and criminal-minded among the gentler sex, the mode of seeking a *quietus* from the ills of life is by drowning themselves.

Although statistical tables have some relative value in an investigation of the subject under consideration, yet they are defective in furnishing *data* as to the causes which induce the chronic or acute forms of suicide. The latter knowledge can only be acquired by an examination into all the antecedents of each case; and then the medical psychologist would obtain positive information both how to prevent or cure these forms of disease, so as to enlighten the public on the subject.

Statistics of suicide are, however, important to indicate what are the particular effects of occupation, of climate, habits of life, age, sex, and so forth. They are also important as correcting much misunderstanding as to the periods of the year that suicide is most prevalent, and the countries in which it is most common. Thus, for example, the names of the following countries are placed in the order of their respective number of suicides to the population — namely, "the United States, England, Prussia, France, Austria, Russia, Italy, and Spain."

The following statistical facts concerning suicide are extracted from page 74 of the Third Report of the Registrar General:—

"This crime, it appears, is most prevalent in London, the proportion being 10·9 to 100,000 inhabitants; next to this discreditable pre-eminence stand the south-eastern counties bordering on the metropolis, where it is 8·4 to 100,000; the range of other parts of England is 6·8 to 7·4, which is the proportion of the western counties, whilst in Wales it is but 2·2. The proportion throughout England and Wales is 6·3, and the total number in the year was 2001."

*The greatest number of suicides occurred in the spring and summer, when crimes attended with violence, and also attacks of insanity, are most common.* Thus, in April, May, and June, there were 563; in July, August, and September, 539; in January, February, and March, 484; and in October, November, and December, 465.

Unfavourable climate cannot, *per se*, specially favour the development of the suicidal tendency. For the climate of Holland is similar to England, and probably even more foggy, and yet its proportion of suicides is less than any of the countries above-mentioned. There must, therefore, be some circumstances capable of modifying the effects of this supposed powerfully predisposing cause; and it is some proof that such is the case, by the fact that the number of suicides in different countries vary considerably at different periods. The common opinion that there are more suicides in the dull months of the year, is refuted by more accurate information. For, on the contrary, it is now proved that a dry hot season fosters a disposition to suicide, and this, for the reason that a dry atmosphere has a tendency to produce a feverish habit; and, as a consequence, tends greatly to increase the irritability and susceptibility of the nervous system. This latter view is founded on accurate observation. We cite the following additional evidence:—

"Of 1131 suicides committed at Berlin, Hamburgh, Westminster, and Paris, there were, in January, February, and March, 237; in April, May, and June, 299; in July, August and September, 335; in October, November, and December, 260.

"A similar influence of the spring and summer weather as favouring the disposition to suicide is proved by the statistics of M. Esquirol, at the Salpêtrière, and by those of M. Prevost, who found that of 113 suicides committed at Geneva in 10 years, the numbers in each month were as follows:—April, 19; June, 17; August, 17; July, 15; October, 14; May, 13; March, 10; November, 9; September, 6; January, 5; February, 5; and December, 5."

The tendency to suicide seems to be least among persons who have out-door employments, and greatest among those of sedentary occupations, whose constitutions are necessarily less robust. The statistical facts which confirm these views are as follows:—

"That there is 1 in 9582 masons, carpenters, and butchers, who committed suicide in one year; and 1 in 1669 tailors, shoemakers, and bakers. The tendency to suicide in those of active out-door occupations being 1 to 5·6 of those of more debilitating and depressing employments."

It may also be noted "that the tendency to suicide is more than twice as great among artisans than it is among labourers—being as 6 in 10,000 of the former, to 2·9 in the same number of the latter."

In the class designated by Mr. Rickman as miscellaneous, "capitalists, bankers, professional and educated persons, the proportion is 4·9 to 10,000."

Mr. Farr does not attribute any great value to the opinion of M. Roué, and other theoretical writers, "that suicide is most common where education is most diffused." Yet he admits that in England suicide is most frequent in the metropolis, the south-eastern, and south-western counties, where the greatest number can write, and is less frequent in Wales, where the proportion of persons who signed the marriage register with a mark is the greatest. He adds, however,—

"There is a general but no constant relation between the state of education thus tested and the commission of suicide. It may be admitted that there is some relation between the development of the intellect and self-destruction; but the connexion must be indirect and accidental. In opposition to the argument derived from agricultural districts and labourers in towns, there is the fact that suicide is more frequent among several classes of artisans than among the better educated people."

This latter view of the subject may be explained, partly as being the result of the excessive habit, among the working-classes alluded to, of taking various kinds of intoxicating beverages. The Registrar adds:—

"If the progress of civilization is to be charged with increase of suicide, we must, therefore, understand by it, the increase of tailors, shoemakers, the small trades, and to the incidental evil to which they are exposed, rather than the advancement of truth, science, literature, and the fine arts."

Subsequently, to show the difference between the influence of education, and the cases where a certain amount of education is occasionally associated, Mr. Farr mentions,

"That about 2·0 in 10,000 persons assured at the Equitable Society, and 7·8 in 10,000 dragoons and dragoon-guards, have been ascertained to commit suicide every year."

In the latter cases, we apprehend, the number is not to be attributed so much to defective education, as it is to their drunken and dissolute habits, to the non-exercise of the moral sentiments, and to the low and degraded condition, generally, of the mental faculties. We cannot attribute suicide to education; if we did so, we might as well say that persons would be likely to have the worst health who rigidly obeyed the laws of mastication, deglutition, and digestion ; and who, besides, would be sure to aggravate the symptoms by their attending to the laws of exercise and ablutions. Every reflecting man will, therefore, form a different inference as to the reason why there is a greater proneness in artisans to commit suicide than those who have out-door occupations, by attributing this difference rather to their breathing so many hours in a vitiated atmosphere, with a liability of vital disturbance from an obstruction of the perspirable exhalations, and other debilitating influences on the nervous system.

Mr. Farr seems to think that in trades least exposed to accidents "the mind is left unexcited by natural dangers, imagines and creates causes of death." But we repeat, that the morbid state which induces suicide in such cases is developed by the want of free air, genial temperatures suitable to the seasons, deficient muscular exercise, gloomy workshops, and uncomfortable homes, and the attempt to counteract these unhealthy and depressing conditions by the excessive use of alcoholic stimuli, until positive injury is induced to the brain and the nervous system. Then restless and irritable, they are rendered incapable of arousing themselves, or of entertaining cheerful trains of thought, and being haunted by the *spectra* of their abnormal minds, they seek relief from the irksomeness and irritation they endure in self-destruction.

In a very excellent article on "Suicide,"* the writer says :—

"It is not proved that education has, as many have asserted, a tendency to increase the number of suicides ; its influence in this respect is not discernible. Neither does the state of poverty or wealth seem to have any material influence. But it is proved that among the inhabitants of large cities suicides are more frequent than in rural districts.

"M. Guerrey has shown, 'Statisque Morale de la France,' that the frequency of suicides in France regularly decreased as the distance of Paris increases. Similar observations were made by Prevost, from statistics at Geneva, and they are completely confirmed by observations made in this country.

"The tendency to suicide is much more frequent in men than in women. By the Westminster returns, in 25 years there were 478 males and 187 females who destroyed themselves : the proportion being as 73 to 27."

We have to notice similar proportions were observed at Geneva, by M. Prevost ; and M. Esquirol, comparing the result of several tables, says, "the proportion may be stated as 3 males to 1 female."

Further details would be of little practical use, but the following proportion of the annual number of suicides in different

* "Penny Cyclopædia."

countries will furnish *data* for grave reflection, and confirm our previous statements, that the results will be found an exact ratio to the degree of departure from the laws of mind and body. M. Quetelet tells us, "that in Russia there is 1 suicide to every 49,182 inhabitants; that in Austria there is 1 in every 20,900; in France, 1 in 18,000; State of Pennsylvania, 1 in 15,875; Prussia, 1 in 14,404; City of Baltimore, 1 to 12,500; and New York, 1 to 7997 inhabitants."

These results would seem to affect the question of mental culture, for in Russia the people are generally ignorant and superstitious, which reduces them to a low intellectual condition; and that at New York, where they are not so, yet there are seven to one suicides in the latter. This would indeed be a gratuitous inference. For the high rate of suicide in the metropolis of the Northern States of America results in consequence of reckless trading, and to the sudden vicissitudes in the prospects and fortunes of such commercial gamblers. Suicide in all ages and countries has been perpetrated either by those whose moral aspirations were obtund, or else by those who reasoned (certainly on false premises) that when life ceased to be pleasurable, that we might get rid of it as we should avoid any other annoyance. And yet in the remotest times in which any opinions on suicide have been recorded, we find some sound views on the subject. We have already quoted the opinions of Cicero, and are tempted to give some few additional statements from the same author. Cicero, "Epis. ad Marium" (Epis. vii.), says, "When he had been defeated (when he laid down his arms under Pompey), there only remained for him to fall into the snare of the enemy, to choose exile, or suicide (mors voluntaria)." But he added, "Cause why I should commit suicide there was *none*, why I should wish it, *much*." Cato destroyed himself at Utica in consequence of the victory of Cæsar in Africa.

Cicero "Tusculan Questiones" xxxiv., 1 and 3, says, "A malis igitur mors abducit non à bonis." This argument was so ably advocated by Hegesias (a philosopher of Cyrene), that Ptolemy forbade him discoursing publicly on the subject, because many people after hearing him committed suicide. Callimichus has an epigram to Cleonbrotius, in consequence of Ambracia, a youth, who having read Plato "On the Immortality of the Soul," threw himself into the sea.*

The discrepant opinions of the ancients on the enormity of self-murder may be judged of by the laws which were enacted for its suppression. A rescript of Hadrian's expressly directed "That those soldiers who, either from impatience of pain or disgust of life, from disease, from madness, from dread of infamy or disgrace,

* *Vide* "Lact. in Aristippi." Va.

had wounded themselves, or otherwise attempted to put a period to their existence, should be punished with 'ignominia.'*

"But the attempt of a soldier on other grounds was a capital offence; or those who, being under prosecution for heinous offences, or being taken in the commission of a great crime, put an end to their existence from fear of punishment, forfeited all their property to the fiscus."†

Suicide was not uncommon among the Romans in the latter days of the Republic, and it became very common under the Emperors, which is evident from the examples given by Tacitus, and by the younger Pliny, who mentions the case of Cornelius Rufus (Epist. xii.), Silius Italicus (cxi., 7), Arria (cxi. 16), and the woman (vi. 24) who succeeded in persuading her husband, who was labouring under an incurable disease, to throw himself, tied to her, into a lake. Except in the cases mentioned in the two tables of the "Digest" above cited, suicide was not forbidden by the Roman law, nor was it discountenanced by public opinion.

Voluntary suicide, by the laws of England, is a crime; and every crime is presumed to be voluntary until the contrary is made apparent. But "the crime of self-murder," or *felonia de se*, are terms calculated to convey a correct notion of the legal character of the offence, or the mode in which it is punished. When such a verdict was pronounced, the personal property was forfeited to the crown. The common law, in the case of suicides, followed the canon law; and if declared guilty of self-murder, were considered to have died in mortal sin, and were interred, and a stake driven through the body. But the law was altered, dispensing with the stake. The self-murdered must now be buried at night, between the hours of nine and twelve, in any church or chapel yard, without the performance of the rites of Christian burial.

The "Code Penal" of France contains no legislation on the subject of suicide.

Of the modern codes of Germany, some adopt the silence of the French code, and others vary in their particular provisions.

In the Bavarian and Saxon codes suicide is not mentioned.

The Prussian code forbids all mutilations of the dead body of a self-murderer, under ordinary circumstances, but declares that it shall be buried without any marks of respect otherwise suitable to the rank of the individual; and it directs that, if any sentence has been pronounced, it shall, as far as feasible, be executed, due regard being had to decency and propriety, on the dead body. Besides which, the body of a criminal who commits self-murder to escape the sentence pronounced against him, is to be buried

* Dig. 49, tit. 16, s. 6, De Re Militari.

† Ibid. 48, tit. 21, s. 31.

at night by the common executioner, at the usual place of executed criminals.

The Austrian code simply provides that the body of the self-murderer shall be buried by an officer of justice, but not in a churchyard, or other place of interments.

From all the evidence submitted, it will be obvious that there has been, and still continues to be, many very discrepant opinions on the subject of suicide, which is regarded rather as a positive criminal tendency, than as a result of some form of morbid affection. It would seem that legislators, philosophers, and jurists, appear to have forgotten that man, in a normal state of mental and bodily health, is strongly influenced by an instinctive love of life, and that when he ceases to experience a sense of self-preservation, it is presumptive proof of some derangement of his mental faculties. It matters little, so far as the argument is concerned, how this state has been induced, whether by animal excesses or moral causes, if, ultimately, there is induced a fixed and determinate notion of self-murder, that the very existence of this unnatural condition is absolute proof of mental indisposition; and although this condition is to be lamented, yet it is contrary to a cultivated humanity to treat the dead body with marks of contumely, just as it would be a want of true benevolence because a drunken man had broken his leg, or otherwise seriously injured himself, to neglect giving him the necessary aid under his suffering, and find an excuse for so doing, that he had himself to blame for it! It is, therefore, not in the province of any community claiming to be civilized and humane, to visit on the suicide impotent penalties, such as the stake, or burying him by the common executioner, &c. These useless punishments to the corpse of the suicide do not prevent others, when their minds are in a similar diseased condition; for if they can inflict on their own living bodies deliberate destruction, how little will they think on what may be inflicted on them when they have neither sensation, perception, nor consciousness!

We therefore affirm that, to prevent suicide, we must endeavour to alter the habits of the people; and, as a discussion on this portion of the subject would necessarily lead to many important details in connexion with education, we shall prefer treating the subject in a separate article. This will be obvious, if we copy a brief extract from Kant's "Elementology of Ethics," as this great metaphysician seems undecided whether to assign actual demerit to the suicide. He says, Aptome i., Chap. 1, "On the Duty owed by Man to himself in respect to his Animal Part:"—

"The first, if not the chiefest duty incumbent on man, in respect of his brute nature, is the self-conservation of his animal estate. The anti-part of this obligation is the deliberate and forethought destruction of his animality; and this may be considered as either total or partial. The total we call murder, and so forth."

Again, on Self-murder, this author says—

"The voluntary divestiture of man's animal part can be called self-murder only when it is shown that such an act is criminal. A crime which may be perpetrated, either singly on our own person, or also at the same time on the person of another—*e.g.*, as when one in pregnancy kills herself. Self-destruction is a crime—murder. Suicide may no doubt be considered as the transgression of a duty owed by any one to his fellow-men—as a violation of the conjugal obligations incumbent upon spouses—as the disregard of the duty owed by a subject to his government (the state); and, lastly, as a dereliction of one's duty to God in this world. But none of these amount to the crime of murder; and the question at present to be considered is, whether or not deliberate self-destruction is in violation of man's duty towards himself, even when abstraction is made from all those other considerations; that is, whether a man ought to acknowledge himself beholden to the self-conservation of his animal part, and beholden so to act, and, too, by force singly of his personality. That a man can injure himself appears absurd (*volenti non fit injuria*); and this was the reason why the Stoics considered it to be the prerogative of a sage to walk with undisturbed soul out of life as out of a smoky room, not urged by any present or apprehended evils, but simply because he could no longer sustain with effect his part in life; and yet this very courage, this strength of soul to advance undauntedly to death, arguing his opinion of somewhat prized by him far higher than life, ought to have taught him not to despoil a being of existence possessing so mighty a mastery and control over the strongest force in his physical system."

He adds something more to the purpose, thus,—

"Mankind, so long as duty is at stake, cannot renounce his personality; that is, by consequence, never, duty being always his incumbent debt; and it is a contradiction to hold that any one were entitled to withdraw himself from his obligations, and to act free, in such sense as need no ground of warrant for his conduct."

We could multiply quotations, but to little practical advantage. The opinions of legislators may be inferred by the laws which apply to suicides, and the theories of philosophers are based upon no certain data; so that, however they may discuss the moral aspect, they appear to be defective in their information as to the causes which may induce the suicidal tendency. Cicero anticipated the true mode of testing the subject, as he affirmed "that a man could not love and hate himself at the same time;" yet this simply states a fact, without giving us its true solution. This could only be obtained by sound physiological and psychological information on the organization of man, the laws which affect it, and which derange the mental faculties; and lastly, to explain what is his normal state, and what constitutes disease, and by distinguishing between what is a temporary disturbance of function and what a permanent affection, in order to assign the degree and the kind of the malady. By these important sciences the student can learn to distinguish between crime and disease, and to indicate the best way of preventing or remedying such affections; thus proving, in the most emphatical manner, the truth of the Baconian aphorism, that "KNOWLEDGE IS POWER!"*

* In this paper we have not mentioned the fact, that the disposition to suicide, like to insanity, are often both hereditary. And in many instances, in our notes of such cases, we have evidence which goes far to prove that there exists a similarity in their history:—that they may remain *latent* for years, until some predisposing circumstance developes their active manifestation. And in both forms of disease, if attention is paid to the hygienic laws, they may be prevented from manifesting any morbid consequences.

